# A Mathematical Model of Aqueous Humor Production and Composition

**DOI:** 10.1167/iovs.63.9.1

**Published:** 2022-08-02

**Authors:** Mariia Dvoriashyna, Alexander J. E. Foss, Eamonn A. Gaffney, Rodolfo Repetto

**Affiliations:** 1 Department of Applied Mathematics and Theoretical Physics, University of Cambridge, United Kingdom; 2 Mathematical Institute, University of Oxford, United Kingdom; 3 Department of Ophthalmology, Nottingham University Hospitals NHS Trust, United Kingdom; 4 Department of Civil, Chemical and Environmental Engineering, University of Genoa, Italy

**Keywords:** aqueous humor, ciliary epithelium, epithelial transport

## Abstract

**Purpose:**

We develop a mathematical model that predicts aqueous humor (AH) production rate by the ciliary processes and aqueous composition in the posterior chamber (PC), with the aim of estimating how the aqueous production rate depends on the controlling parameters and how it can be manipulated.

**Methods:**

We propose a compartmental mathematical model that considers the stromal region, ciliary epithelium, and PC. All domains contain an aqueous solution with different chemical species. We impose the concentration of all species on the stromal side and exploit the various ion channels present on the cell membrane to compute the water flux produced by osmosis, the solute concentrations in the AH and the transepithelial potential difference.

**Results:**

With a feasible set of parameters, the model predictions of water flux from the stroma to the PC and of the solute concentrations in the AH are in good agreement with measurements. Key parameters which impact the aqueous production rate are identified. A relevant role is predicted to be played by cell membrane permeability to ${\text{K}}^{+}$ and ${\text{Cl}}^{-}$, by the level of transport due to the Na^+^-H^+^ exchanger and to the co-transporter of Na^+^/K^+^/2Cl^−^; and by carbonic anhydrase.

**Conclusions:**

The mathematical model predicts the formation and composition of AH, based on the structure of the ciliary epithelium. The model provides insight into the physical processes underlying the functioning of drugs that are adopted to regulate the aqueous production. It also suggests ion channels and cell membrane properties that may be targeted to manipulate the aqueous production rate.

Aqueous humor (AH) formation has been an important topic in ocular research for several decades, owing to its fundamental role in regulating IOP and in the management of glaucoma. AH is an aqueous solution containing a mixture of electrolytes, organic solutes, growth factors, and select proteins^1^ that fills the posterior and anterior chambers (PC and AC) of the eye (Fig. 1). It is well-established that AH is produced by the ciliary epithelium (CE) at a rate of 1 to 3 µL/min^2^^,^^3^; it flows from the PC into the AC through the pupil and exits the eye via the conventional and uveoscleral pathways.^4^ The balance between the rate of AH production and resistance to its drainage governs the IOP, which typically ranges between 12 and 22 mm Hg, in healthy subjects. An elevated IOP is correlated with the occurrence of glaucoma.^5^^,^^6^ Decreasing the AH production rate is one of the possible strategies to lower the IOP and treat glaucoma.

**Figure 1.**
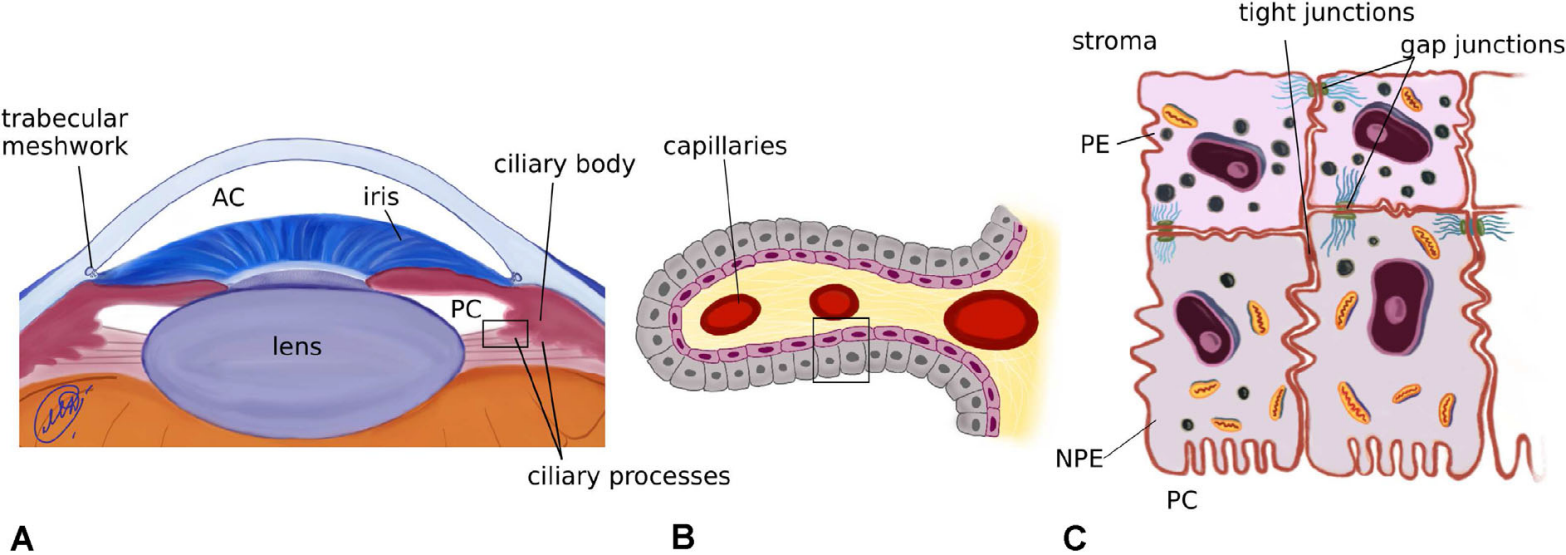
Sketches of a cross section of the human eye (**A**), of the ciliary processes (**B**) and of the CE (**C**).

The CE is a bilayer of cells that consists of the pigmented epithelium (PE), facing the stroma, and the non-pigmented epithelium (NPE), facing the PC (Fig. 1(B)). Selective tight junctions that connect NPE cells separate the stromal side from the PC side. The two cell layers are connected by gap junctions, effectively forming a functional syncytium (see Fig. 1(C)). Therefore, the epithelium behaves like a secretory monolayer epithelium. This view is reinforced when one considers the distribution of ion channels and transporters, and of the ${\text{Na}}^{+}$-${\text{K}}^{+}$ pump, as shown in Figure 2, where the NPE basolateral membrane has a channel distribution reminiscent of an epithelial apical domain.^7^^–^^10^

**Figure 2.**
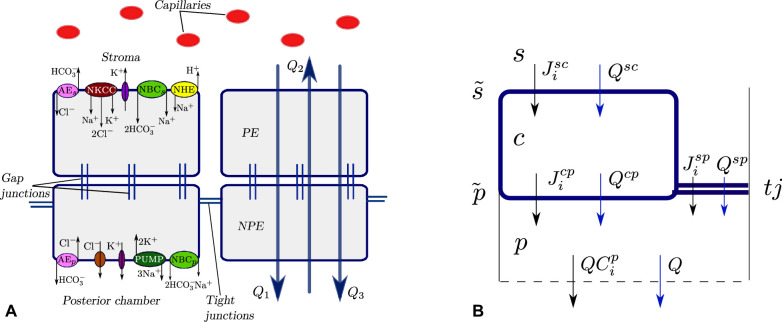
(**A**) Ion channels in the CE. PE basolateral membrane: nkcc: co-transporter of ${\text{Na}}^{+}$/${\text{K}}^{+}$/2${\text{Cl}}^{-}$; ${ae}_{s}$: ${\text{Cl}}^{-}$-${\text{HCO}}_{3}^{-}$ exchanger; nhe: ${\text{Na}}^{+}$-${\text{H}}^{+}$ exchanger; $nbc_{s}$: ${\text{Na}}^{+}$- ${\text{HCO}}_{3}^{-}$ cotransporter, presumed to be of stoichiometry 1:2; ${\text{K}}^{+}$- channels. NPE basolateral membrane: pump: ${\text{Na}}^{+}$-${\text{K}}^{+}$ ATPase; $ae_{p}$: ${\text{HCO}}_{3}^{-}$-${\text{Cl}}^{-}$ exchanger; $nbc_{p}$: ${\text{Na}}^{+}$- ${\text{HCO}}_{3}^{-}$ cotransporter; ${\text{K}}^{+}$ and ${\text{Cl}}^{-}$ channels. (**B**) A sketch of the compartmental model with water and ion fluxes (not to scale). The symbols s, c and p denote stroma, intracellular space and PC respectively. The symbols $\tilde{p},\;\tilde{s}$ and tj denote the PC and stromal cell membranes and the tight junction. In the direction from region m to region k, $J_{i}^{mk}$ denotes flux of solute i and $Q^{mk}$ the water flux (see main text).

The components of the AH are ultimately derived from the blood and they have to traverse the endothelium of the ciliary circulation to enter the stroma, by a process of ultrafiltration, cross from the stroma to enter the CE, passing through the basolateral surface of the PE, and cross from the CE to enter the PC, crossing the basolateral surface of the NPE. This article addresses these last two steps, to develop a model that can represent these processes.

Mathematical modeling has proven to be useful in understanding ocular fluid flows.^11^ A successful model allows one to explain a particular phenomenon, separate the underlying mechanisms, determine their relative importance and make testable predictions. In the context of AH formation, a mathematical model would be useful to understand the key mechanisms governing AH production rate and suggest possible targets for future drug development.

Water transport across the CE involves several possible mechanisms. The first is a mechanical pressure difference, Δp, between the stroma and the PC that induces a water flux into the PC. The second is an oncotic pressure difference, $\Delta \Pi_{p}$, with the oncotic pressure being higher on the stromal side. This drives fluid from the PC towards the stroma. The conventional view is that these two contributions approximately balance each other out.^12^^,^^13^ The main mechanism is thought to be osmotic pressure difference $\Delta \Pi_{s}$ between the two sides of the CE, generated by active transport of ions, which produces a net flow towards the PC. In mathematical terms, such mechanisms of AH production are expressed by the Starling law:

(1) $$\begin{array}{c} q=K\left(\Delta p-\sigma_{p}\Delta \Pi_{p}-\sigma_{s}\Delta \Pi_{s}\right).\; \end{array}$$

In this expression, q is water flux per unit surface area (with the dimensions of velocity), K is the hydraulic conductivity of the CE, and $\sigma_{s}$ and $\sigma_{p}$ are reflection coefficients for ions and proteins, respectively. Oncotic and osmotic pressures can be evaluated using van't Hoff's law ΔΠ=RTΔC, with ΔC being difference in solute concentration (osmolarity) across the cell layer, R the universal gas constant, and T the absolute temperature. Equation (1) has been used in several modeling works to evaluate AH production.^14^^,^^15^ However, with this approach the distribution of ion channels and transporters present on the cell membrane is completely disregarded and thus it is not possible to obtain information about their specific role in generating the osmolarity jump across the CE.

In a recent work, Sacco et al.^16^ proposed a more detailed model that takes into account the distribution of ion channels on the cell membrane (with the exception of the ${\text{Na}}^{+}$-${\text{HCO}}_{3}^{-}$ channel, which was omitted). The authors impose the concentration of all species both on the stromal and PC sides of the CE and use the model to compute the concentration of all species in the cell. This implies that the model cannot predict how AH production rate would be modified by inhibition of some of these channels, since the osmolarity jump across the CE is, again, prescribed.

In the present article, we propose an alternative approach that allows us to predict not only ion and water transport, but also AH composition in the PC, given the concentrations of all species in the stroma. Because in our model fluxes and concentrations depend on the distribution of channels and transporters on the cell membranes, we can predict how they would react to the inhibition of specific channels. This is of practical and conceptual importance, because the inhibition of specific channels is the aim of certain drugs used to manipulate the AH production rate.

## Mathematical Model

In this work, we propose a compartmental 0-dimensional model of the transport of water and chemical species across the CE, a schematic of which is presented in Figure 2B. We consider three regions: the stroma (denoted with superscript s), the cellular compartment, which encompasses both NPE and PE cells (c) and the PC (p). The compartments are separated by the stromal membrane (denoted with superscript $\tilde{s}$) and the PC membrane ($\tilde{p}$). We further assume that the tight junctions act as semi-permeable membranes between stroma and the PC (tj). The spatial variation within the compartments is neglected and all variables are averaged over their respective domains. This assumption is not valid in the cleft gap between two adjacent cells, where the spatial variation of ion concentration is a key ingredient for generating standing gradient osmotic flow.^17^ However, in this work we only consider osmotic flow driven by the difference in ion concentration between the PC and the stroma and neglect possible local osmosis in the cleft gap, as further commented upon in the discussion.

We consider that all domains contain an aqueous solution of seven different species: ${\text{Na}}^{+}$, ${\text{K}}^{+}$, ${\text{Cl}}^{-}$, ${\text{HCO}}_{3}^{-}$ (bicarbonate), ${\text{H}}^{+}$, ${\text{CO}}_{2}$, and ${\text{H}}_{2}{\text{CO}}_{3}$ (carbonic acid). We denote the concentrations of these solutes as $C_{i}^{m}$ (mM), where subscript i denotes the species (i=1,⋯,7, enumerated in the order above) and superscript m the region (m=s,c,p). We denote their valence and charge with $z_{i}$. The cell contains fixed non-diffusible charged solutes, which we denote with X, and their concentration and valence are denoted as $C_{X}^{c}$ and $z_{X}$. The electric potential in region m=s,c,p is denoted with $V^{m}$.

Solutes are transported across the membranes of the domain via ion channels, co-transporters, and exchangers and we indicate a flux of a solute i from region m to region k with $J_{i}^{mk}$ (mol/s). For instance, the flux of ${\text{Na}}^{+}$ from the stroma into the cell is named $J_{1}^{sc}$. We consider that the stromal side of the membrane contains co-transporters of ${\text{Na}}^{+}$, ${\text{K}}^{+}$and 2${\text{Cl}}^{-}$(denoted with nkcc), anion exchangers of ${\text{Cl}}^{-}$ and ${\text{HCO}}_{3}^{-}$ ($ae_{s}$),^7^ ${\text{Na}}^{+}$-${\text{H}}^{+}$ exchangers (nhe),^8^ co-transporters of ${\text{Na}}^{+}$ and 2${\text{HCO}}_{3}^{-}$ ($nbc_{s}$) and ${\text{K}}^{+}$ channels,^18^ as shown in Figure 2A. The PC side of the membrane has ${\text{Na}}^{+}$-${\text{K}}^{+}$ ATPase (denoted with pump),^9^ ${\text{Cl}}^{-}$-${\text{HCO}}_{3}^{-}$ exchangers ($ae_{p}$), ${\text{Na}}^{+}$-${\text{HCO}}_{3}^{-}$ co-transporters ($nbc_{p}$), ${\text{K}}^{+}$ and ${\text{Cl}}^{-}$ channels.^18^^,^^19^ The tight junctions are assumed to be permeable to ${\text{Na}}^{+}$, ${\text{K}}^{+}$, ${\text{Cl}}^{-}$ and ${\text{HCO}}_{3}^{-}$. In addition, all membranes and the tight junction are permeable to ${\text{CO}}_{2}$ and ${\text{H}}_{2}{\text{CO}}_{3}$.

The expressions for the solute fluxes (mol/s) across each transporter are reported in the Appendix, but they all have a common representation as the flux through channel k can be formally written as

(2) $$\begin{array}{c} J_{k}=AP_{k}f\left(C,V\right),\; \end{array}$$

with f being a non-linear, dimensionless function that involves solute concentrations C and potentials V on both sides of the membrane, $P_{k}$ the intensity of channel transport (mol/m${}^{2}$/s), and A (m${}^{2}$) the surface area of the considered membrane.

The flux of solute i can also be driven across the membrane between regions m and k by electrodiffusion through ion channels (or by diffusion for non-charged solutes), and has the generalized representation

(3) $$\begin{array}{c} J_{i}^{mk}=APg\left(C,V\right),\; \end{array}$$

where P is a membrane permeability to this solute (m/s) and g(C,V) is a function that has dimensions of concentration (mM), with a dependence on solute concentrations C and potentials V either side of the membrane (see the Appendix for details).

The flux of each solute i from region m to region k, $J_{i}^{mk}$, is the sum of fluxes through all channels and transporters on that membrane, which involve solute i. We also assume that at the outlet of the PC the solute is only driven by advection ($QC_{i}^{p}$, with Q [m${}^{3}$/s] being the rate of aqueous production) and that diffusive flux can be neglected. Indeed, the velocity in thin iris-lens channel is about U≈0.2 mm/s,^4^ so using typical PC length H≈6 mm and a diffusion coefficient $D\approx 2\cdot 10^{-3}$ mm${}^{2}$/s,^17^ we can calculate the Péclet number, Pe=UH/D≈600, confirming that advection has a dominant contribution at the PC exit.

The considered solutes interact through the following chemical reactions

(4) $$\begin{array}{c} {\text{HCO}}_{3}^{-}+{\text{H}}^{+}\overset{k_{1}}{\underset{k_{-1}}{⇌}}{\text{H}}_{2}{\text{CO}}_{3}\overset{k_{d}}{\underset{k_{h}}{⇌}}{\text{CO}}_{2}+{\text{H}}_{2}\text{O},\; \end{array}$$

with $k_{1}$, $k_{-1}$, $k_{d}$, and $k_{h}$ being the reaction rate constants. The first reaction is the dissociation of ${\text{H}}_{2}{\text{CO}}_{3}$ into the ions ${\text{HCO}}_{3}^{-}$ and ${\text{H}}^{+}$, which is rapid and happens almost instantaneously. The second reaction is normally slow, but can be catalyzed up to six orders of magnitude in speed by carbonic anhydrase (CA).^20^ CA II and IV are present in the NPE cells.^10^ We denote with $R_{i}^{m}$ the reaction terms (production minus consumption) of species i in region m, which we model with the mass action law^21^ (see Appendix).

Water is transported across each membrane by osmosis and we denote the water flux from region m to region k with $Q^{mk}$ (m${}^{3}$/s) and it has the following generic expression

(5) $$\begin{array}{c} Q^{mk}=-AK\sigma RT\Delta C^{mk},\; \end{array}$$

where $\Delta C^{mk}$ is the difference in osmolarity between the two sides of the membrane, K (m/s/Pa) is hydraulic conductivity, and A is the surface area of the membrane. For example, water flux from stroma to the cell can be written as $Q^{sc}=-A^{\tilde{s}}K^{\tilde{s}}\sigma RT\left[\sum_{i=1}^{7}\left(C_{i}^{s}-C_{i}^{c}\right)-C_{X}\right]$.

In the stromal compartment all species concentrations $C_{i}^{s}$ and the electric potential are imposed.

We now may finally write the steady state conservation of mass in the cells and the PC as follows

(6a) $$\begin{array}{c} \begin{array}{cc} \text{Cell} & \text{PC}\\ J_{i}^{sc}-J_{i}^{cp}+R_{i}^{c}=0, & J_{i}^{cp}+J_{i}^{sp}-QC_{i}^{p}+R_{i}^{p}=0, \end{array}\; \end{array}$$

(6b) $$\begin{array}{c} Q^{sc}-Q^{cp}=0,\;Q^{cp}+Q^{sp}-Q=0,\; \end{array}$$

with i=1,⋯,7. These equations are simplified by taking into account the fact that the first step of reaction (4) is almost instantaneous (please refer to the Appendix for details). The equations are complemented by electroneutrality in both the cell and the PC

(6c) $$\begin{array}{c} \sum_{i=1}^{7}z_{i}C_{i}^{c}+z_{X}C_{X}=0,\;\sum_{i=1}^{7}z_{i}C_{i}^{p}=0.\; \end{array}$$

In total, we have 18 nonlinear algebraic equations for an equal number of unknowns: concentrations in the cell and the PC: $C_{i}^{c}$, $C_{i}^{p}$ (14 in total), X, water flux Q, and potentials in the cell and the PC $V^{c}$, $V^{p}$. These equations are solved in Matlab using the function solve for symbolic variables and the stability of the solution was verified by solving a time-dependent version of the model, using the Matlab function ODE45, which is based on an explicit Runge-Kutta (4,5) formula.^22^

Model parameters are reported in Table 1 and discussed in detail along with other model parameters in the Appendix.

**Table 1.** Model Parameters.


Geometrical parameters [**GP**]											
Parameter	L (m)		H (m)		$A^{\tilde{s},\tilde{p}}$ (m${}^{2}$)		$A^{tj}$ (m${}^{2}$)		$A^{PC}$ (m${}^{2}$)		
Value	$10^{-5}$		6$\cdot 10^{-3}$		$6\cdot 10^{-4}$		$6\cdot 10^{-7}$		$2.6\cdot 10^{-5}$		

Hydraulic conductivities [**P-H2O**] and permeabilities to ${\text{CO}}_{2}$ and ${\text{H}}_{2}{\text{CO}}_{3}$ [**P-C**]											
Parameter	$K^{\tilde{s},tj}$ (m/s/Pa)	$K^{\tilde{p}}$ (m/s/Pa)		$P_{co_{2}}^{\tilde{s},tj}$ (m/s)		$P_{co_{2}}^{\tilde{p}}$ (m${}^{2}$)		$P_{h_{2}co_{3}}^{\tilde{s},tj}$ (m/s)		$P_{h_{2}co_{3}}^{\tilde{p}}$ (m/s)	
Value	$2\cdot 10^{-11}$	$2\cdot 10^{-10}$		$1.5\cdot 10^{-3}$		$1.5\cdot 10^{-2}$		$1.28\cdot 10^{-5}$		$1.28\cdot 10^{-4}$	

Reaction rates [**RR**] and other parameters [**Other**]											
Parameter	$k_{d}$ (1/s)	$k_{h}$ (1/s)		$K_{d}=k_{1}/k_{-1}$ (1/mM)		T (K)		σ		$z_{X}$	
Value	$4.96\cdot 10^{5}$	$1.45\cdot 10^{3}$		5.3		310		1		-1.5	

Ion channels [**P-ions**]											
Parameter	$P_{pump}$	$P_{n}kcc$	$P_{ae_{s}}$	$P_{ae_{p}}$	$P_{nbc_{p}}$	$P_{nbc_{s}}$	$P_{n}he$	$P_{{\text{K}}^{+}}^{\tilde{s}}$	$P_{{\text{K}}^{+}}^{\tilde{p}}$	$P_{{\text{Cl}}^{-}}^{\tilde{p}}$	$P^{tj}$
Value	6	1	4	0.4			3.4	5	30	6	600
Equation	(15)	(9)	(12)	(13)	(11)	(10)	(14)	(8)			

Each section corresponds to a certain parameter group, each having its own marker reported in square brackets, which is used in Appendix 2 for easier referencing. For example, the reaction rates are referred to as **RR**. In the last section we report permeabilities and intensities of the ion channels used in the model. Left Side: Intensities associated with the fluxes through ion transporters as in (2), expressed in $\cdot 10^{-6}$ mol/m${}^{2}$/s. Right Side: Permeability of ion channels in $\cdot 10^{-8}$ m/s as in (8). In last row we report the number of the mathematical expression of the fluxes where these parameters appear. Justification for the choice of some of these parameters and the corresponding sources are reported in Appendix 2.

### Global Sensitivity Analysis

We used a global sensitivity analysis owing to the large number of parameters involved. This strategy allowed us to identify those that affect the model results the most. In particular, we used a variance-based method (extended Fourier amplitude sensitivity test [eFAST]), proposed in Saltelli et al.^23^ and adapted to biological systems by Marino et al.^24^ *Inter alia,* eFAST produces a dimensionless total sensitivity index for each parameter, which estimates the variance associated with the variation of the parameter, including its interactions with the other parameters. In simple terms, the greater the value of the index for a given parameter the higher its influence on the model result under consideration. All parameters considered in the sensitivity analysis were checked to be structurally identifiable using the scaling method proposed by Castro and de Boer.^25^

## Results

### Baseline Values

We first show the results obtained running the model with the ‘baseline’ parameters values, estimated as detailed in the Appendix and summarized in Table 1.

In the first part of Table 2 (Experimental measurements), we report available experimental measurements from the literature of the concentrations of various solutes and of the electric potentials. We show the corresponding values predicted by the model in the second section of the table (Model - baseline). Note that model variables in the stromal compartment are prescribed, and have been slightly adjusted with respect to the experimental ones, as the latter do not satisfy the electroneutrality condition, which is one of the assumptions underlying the model and a fundamental physical constraint away from Debye layers. All concentration values predicted by the model in the cell and PC are in good quantitative agreement with experiments and so is the transepithelial potential difference ($V^{p}$).

**Table 2.** Concentrations of Ions, pH, and the Potential, in the Cell, Stroma, and the PC.

	Experimental measurements^26^^,^^27^^,^^18^^,^^7^					
	[${\text{Na}}^{+}$] (mM)	[${\text{K}}^{+}$] (mM)	[${\text{Cl}}^{-}$] (mM)	[${\text{HCO}}_{3}^{-}$] (mM)	pH	V (mV)

Stroma	148	4	107	26		0
Cell	15 ± 3	162 ± 14	46 ± 5			−70
PC	152	3.9	131	22		−1
	Model - baseline					
	[${\text{Na}}^{+}$] (mM)	[${\text{K}}^{+}$] (mM)	[${\text{Cl}}^{-}$] (mM)	[${\text{HCO}}_{3}^{-}$] (mM)	pH	V (mV)

Stroma	150	5	130	25	7.42	0
Cell	17.8	154.4	45	26.9	7.46	−75.6
PC	151.8	4.3	126.9	28.6	7.49	−1.56
	Model - baseline, no CA					
	[${\text{Na}}^{+}$] (mM)	[${\text{K}}^{+}$] (mM)	[${\text{Cl}}^{-}$] (mM)	[${\text{HCO}}_{3}^{-}$] (mM)	pH	V (mV)

Cell	5.35	165.3	59.7	18.3	8.78	−73
PC	135	20.3	145	10	7.04	−22

In Table 3, we report ion and water fluxes. With a baseline set of parameters, the model is capable of predicting the AH formation rate (i.e., the flux from the stroma into the PC) with the correct order of magnitude. The model predicts that all the ion fluxes are directed towards the PC; ${\text{Cl}}^{-}$ flux has a magnitude compatible with measurements, whereas the ${\text{Na}}^{+}$ flux is overestimated, which is considered further in the Discussion.

**Table 3.** Fluxes of Ions and Water From the Literature and Predicted by the Model.

Fluxes	${\text{Na}}^{+}$	${\text{K}}^{+}$	${\text{Cl}}^{-}$	${\text{HCO}}_{3}^{-}$	H${}_{2}$O
Units	µmol/m${}^{2}$/s				m${}^{3}$/s
Experiments	0.932^28^		[2.8,7.86]^29^^,^^30^		$5\cdot 10^{-11}$ ^3^
Model baseline	7.34	0.21	6.16	1.38	$2.91\cdot 10^{-11}$
Model baseline, no CA	3.61	0.54	3.87	0.28	$1.6\cdot 10^{-11}$

### Sensitivity Analysis

In the previous section, we have shown that with the ‘baseline’ set of parameter values the model satisfactorily reproduces experimental observations, though noting the above-mentioned discrepancy with the ${\text{Na}}^{+}$ flux. In this section, we use a global sensitivity analysis, to identify the intensities of ion fluxes and permeabilities of ion channels that affect the model results the most.

Ion and water fluxes originate from the active pumping of the ${\text{Na}}^{+}$-${\text{K}}^{+}$ ATPase, thus the pump intensity obviously influences all the model results. Indeed, reduction of the parameter $P_{pump}$ (see Equation (2)) by 50% resulted in average decrease of AH secretion by about 25% (results not shown).

**Figure 3.**
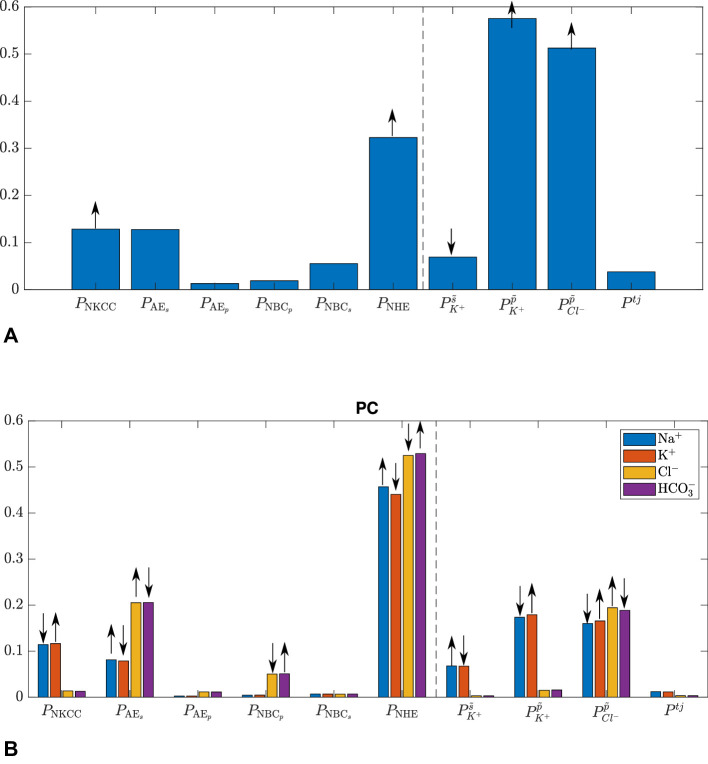
Total sensitivity index for (**A**) production rate Q and (**B**) concentrations in the PC. The arrows indicate how the given parameter influences the model output and are based on the total sensitivity index, which is an output of the eFAST method.^23^ The vertical dashed line separates the intensities of ion channels, $P_{k}$ in Equation (2), from the permeabilities P in Equation (3).

In the sensitivity analysis reported in Figure 3, we fix the intensity of the ${\text{Na}}^{+}$-${\text{K}}^{+}$ ATPase pump and vary the intensities of the other channels and transporters in Table 1 (last section, [**P-ions**]), for a total number of ten parameters. We vary each parameter within ±50% of the value in Table 1 and we perform 5973 model runs for each varied parameter, resulting in 59,730 simulations in total. The way the model parameter space is spanned is chosen by the eFAST method itself, given sampling parameters. In particular, the values of all model parameters are varied within their respective range harmonically, each one at a specific frequency.^23^ We study their effect on AH production rate (Fig. 3A and on the concentrations of ${\text{Na}}^{+}$, ${\text{K}}^{+}$, ${\text{Cl}}^{-}$ and ${\text{HCO}}_{3}^{-}$ in the PC (Fig. 3B). In the figure the bars height indicates the value of the total sensitivity index; the arrows at the top of each bar show how the given parameter influences the model output: an upwards pointing arrow indicates that the model output under consideration increases as the parameter is increased. We note that we have also varied parameters in a range of ±25% and ±75% of the baseline values of Table 1, with no substantive qualitative changes in the results.

Figure 3A shows that AH production rate is very sensitive to the permeability to ${\text{K}}^{+}$($P_{K^{+}}^{\tilde{p}}$), and ${\text{Cl}}^{-}$($P_{Cl^{-}}^{\tilde{p}}$) and to the intensities of the ${\text{Na}}^{+}$-${\text{H}}^{+}$exchanger ($P_{n}he$) and the co-transporter of ${\text{Na}}^{+}$/${\text{K}}^{+}$/2${\text{Cl}}^{-}$ ($P_{n}kcc$). The intensity of the nhe also has significant influence on ion concentrations in the AH (Fig. 3B).

### Role of CA Inhibition

In this section, we investigate how CA inhibition modifies the model predictions. We recall that CA catalyzes the second reaction in Equation (4), which in the absence of CA is slow. In our model, we impose CA inhibition by reducing reaction rates $k_{d}$ and $k_{h}$ in reaction (4) by a factor of $10^{6}$.^20^

**Figure 4.**
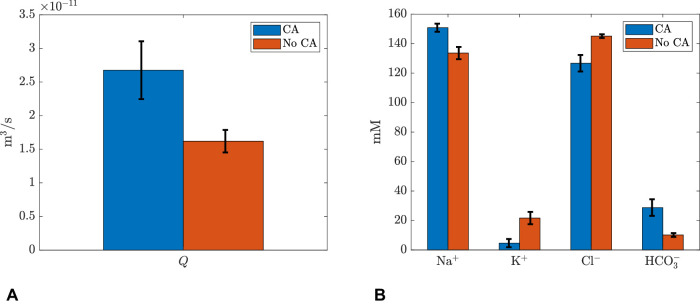
Change of (**A**) production rate Q and (**B**) concentrations in the PC with CA inhibition.

**Figure 5.**
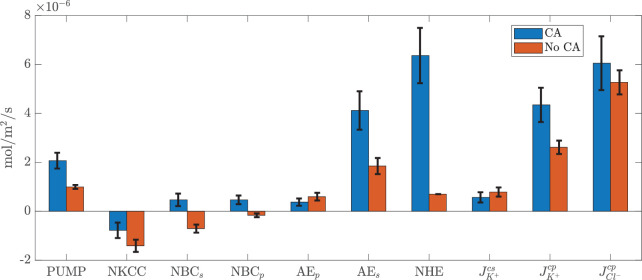
Change of ion fluxes through each individual channel with CA inhibition (see Appendix for detailed expression of ion channel fluxes). The value is positive if the flux is directed outside the cell. For anion exchangers, the value is positive if ${\text{HCO}}_{3}^{-}$ goes out of the cell and flux through nhe is positive if ${\text{H}}^{+}$ is transported out of the cell.

Results for baseline parameters except that CA is inhibited are reported in Tables 2 and 3 Model baseline - no CA. The ${\text{HCO}}_{3}^{-}$ concentration in the PC drops significantly and ${\text{K}}^{+}$concentration increases. The water flux is decreased by approximately 45%. We then perform a parameter variation similar to the one described for sensitivity analysis, but with inhibited CA. The results are reported in Figures 4 and 5, where blue bars refer to the normal case and orange bars to CA inhibition. In the figures, bar height is computed by averaging all values obtained from parameter variation and error bars represent the corresponding standard deviation. In Figure 4A, we show that the model reproduces the reduction of aqueous production as a response to CA inhibition by 40% on average, which is in agreement with experimental observations.^31^ The model also predicts an increase of ${\text{K}}^{+}$ and ${\text{Cl}}^{-}$concentration in the PC and a corresponding decrease in ${\text{Na}}^{+}$ and ${\text{HCO}}_{3}^{-}$.

Finally, Figure 5 reports the variation of all ion fluxes through each individual channel. We note that nhe is decreased by about six times with inhibition of CA. Furthermore, nbc on both membranes reverse their transport direction, owing to a change in concentration of ${\text{HCO}}_{3}^{-}$ in the cell and the PC.

## Discussion and Conclusions

We have presented a mathematical model that successfully captures many of the observed features of aqueous production. The model explains AH production on the basis of osmosis induced by selective ion pumping across the CE. This is consistent with the standard view that the contributions of oncotic and hydrostatic pressure differences, are both small and of opposite sign, and so cancel each other out.^12^^,^^13^

We account for the presence of seven different solutes (${\text{Na}}^{+}$, ${\text{K}}^{+}$, ${\text{Cl}}^{-}$, ${\text{HCO}}_{3}^{-}$, ${\text{H}}^{+}$, ${\text{CO}}_{2}$, and ${\text{H}}_{2}{\text{CO}}_{3}$) that can chemically interact. We impose the concentrations of all species in the stroma (essentially a fixed boundary condition) and compute water and ion fluxes across the CE together with AH composition. This process is a significant improvement on the previous mathematical models of AH production, where the species concentrations both at the stroma and PC were prescribed. The advantage of our approach is that the model can now predict how inhibition of a specific channel would modify AH production rate and composition.

We identify a ‘baseline’ set of parameter values, with most of the parameters being estimated on the basis of experimental measurements. With this set of parameters, the model predicts the generation of a AH flux from the stroma into the PC. This flow has the correct order of magnitude, although it slightly underestimates the experimental measurements (Tables 2 and 3). This outcome might be a consequence of the fact that we neglected the role of the cleft gaps among adjacent cells in producing a standing gradient osmotic flux, as described by Diamond and Bossert.^32^

With the baseline set of parameters, the model predicts concentrations in the cell and the PC very similar to those measured experimentally (Table 3). Ion fluxes are predicted to be directed towards the PC, which confirms the generally accepted paradigm that “water follows the ions.” The ${\text{Cl}}^{-}$ flux has a magnitude in line with measurements, whereas the ${\text{Na}}^{+}$ flux is overestimated (Table 2). We note, however, that in the measurement of ${\text{Na}}^{+}$ current,^28^ the authors detected only about 25% of ${\text{Na}}^{+}$channels,^18^ which suggests that the measured flux may be about four times higher than that reported in Table 2, resulting in the value of $3.72\cdot 10^{-6}$ mol/m${}^{2}$/s, which is the same order of magnitude as our prediction.

Experimental evidence shows that the inhibition of the ${\text{Na}}^{+}$-${\text{K}}^{+}$ ATPase markedly increases the intracellular ${\text{Na}}^{+}$ content, and reduces the intracellular ${\text{K}}^{+}$ content,^26^ although the authors do not report quantitative results. This behavior is also reproduced by our model (not shown in the figures).

We used a global sensitivity analysis to study the influence of each model parameter on the predicted AH production rate and AH chemical composition. We found that the AH production rate is very sensitive to the cell membrane permeabilities to ${\text{K}}^{+}$ and ${\text{Cl}}^{-}$ and to the intensities of the nhe exchanger and of the nkcc co-transporter. The properties of nhe also have a significant influence on ion concentrations in the AH.

Because the IOP is the result of a balance between AH production rate and resistance to its drainage, one of the possible strategies to decrease the IOP is to use drugs that act on AH production rate. Avila et al.^33^ showed that topical application of different direct blockers of the nhe exchanger produce IOP reduction in mice, arguably secondary to a reduction in AH production. CA inhibitors are among the most effective drugs used to decrease the rate of AH production; for instance, CA inhibits water flux and results in reduction of water flux by 21%-41%.^31^ We studied with the model the effect of inhibiting CA and found that this leads to a significant decrease in AH production rate, in agreement with clinical observations. Thus, the model provides a sound physical basis to explain the role of CA on AH production. Furthermore, Counillon et al.^8^ explained the effect of CA inhibition on AH formation through the reduction of ${\text{H}}^{+}$ and ${\text{HCO}}_{3}^{-}$ concentrations, which leads to reduction of fluxes through NHE-1 and AE2 channels. This finding is confirmed by our model predictions and supports this explanation for the effect of CA.

The mechanisms underlying AH production are extremely complicated and the model proposed only focuses on some aspects and we deliberately neglect effects that likely have some influence, but would complicate the picture significantly. Among the most important limitations of our model is that we neglect interactions with blood flow. Kiel et al.^13^ proposed a lumped parameter mathematical model that couples blood flow in the choroid and ciliary processes (including autoregulation mechanisms), oxygen delivery to and consumption by ocular tissues, and AH production. They obtain results in agreement with experimental observations that highlight the interplay between ciliary blood flow and AH production.

Another limitation of our model is that it does not account for local osmosis, that is, water transport generated as a consequence of concentration gradients within the cleft gaps among cells.^32^ This mechanism was found to be by far the dominant mechanism inducing water transport across the retinal pigment epithelium,^17^^,^^34^ and it is likely to influence AH production. Nonetheless, although local osmosis is fundamental for water transport across the retinal pigment epithelium because there is no osmolarity jump across this cell layer, such an osmolarity jump exists across the CE and is correctly predicted by our model. Thus, the role of local osmosis is relatively diminished, justifying the use of a zero-dimensional lumped parameter model without the mechanism of local osmosis.

## Appendix A Appendix 1

### Mathematical Description of Ion Channels

In this section, we report the mathematical expressions used to model ion fluxes through the ${\text{Na}}^{+}$-${\text{K}}^{+}$ ATPase pump, ion channels and cotransporters as generalized in Equations (2) and (3).

#### Non charged Solute

We start with the non-charged solutes: ${\text{CO}}_{2}$ and ${\text{H}}_{2}{\text{CO}}_{3}$ (i=6,7). Fluxes of these species across the membrane are modeled using an expression derived from the integration of the diffusion equation across the membrane,^35^

(7) $$\begin{array}{c} J_{i}^{mk}=AP\left(C_{i}^{m}-C_{i}^{k}\right),\; \end{array}$$

where A (m${}^{2}$) is the surface area of the membrane and P (m/s) the permeability of the membrane. This expression is used to model the fluxes of ${\text{CO}}_{2}$ and ${\text{H}}_{2}{\text{CO}}_{3}$ across each membrane and the tight junction.

#### Ion Channels

Ion channels, such as ${\text{K}}^{+}$ channels, are selective and transport single ions down their electrochemical gradient. A frequently used expression for the membrane flux due to these channels is the Goldman-Hodgkin-Katz,^35^ according to which the flux of ion i from region m to region k can be written as

(8) $$\begin{array}{c} J_{i}^{mk}=APz_{i}\phi^{mk}\frac{C_{i}^{m}-C_{i}^{k}\exp\left(-z_{i}\phi^{mk}\right)}{1-\exp\left(-z_{i}\phi^{mk}\right)},\; \end{array}$$

where factor P is a membrane permeability to this ion (m/s), A is the area of the membrane, $\phi^{mk}=F\left(V^{m}-V^{k}\right)/RT$ is the dimensionless potential difference across the membrane, F is Faraday's constant, R the universal gas constant, and T is temperature. This expression is used to model the flux through ${\text{K}}^{+}$ channels on the cell membrane, ${\text{Cl}}^{-}$ channels on the PC side of the membrane and ion fluxes across the tight junction.

The expressions (7) and (8) are generalized in Equation (3).

#### Cotransporters and Antiporters

Cotransporters, such as ${\text{Na}}^{+}$-${\text{K}}^{+}$-${\text{Cl}}^{-}$ (nkcc), and antiporters are passive channels and perform joint transport of solutes. We model these fluxes as a function of the difference in overall chemical potential across the membrane, an approach that is valid in a near-equilibrium system.^36^

Following this approach the flux out of the cell of the nkcc channel can be written as

(9) $$\begin{array}{c} J_{n}kcc=A^{\tilde{s}}P_{n}kcc\ln\left(\frac{C_{K}^{c}C_{\mathrm{Na}}^{c}{\left(C_{\mathrm{Cl}}^{c}\right)}^{2}}{C_{K}^{s}C_{\mathrm{Na}}^{s}{\left(C_{\mathrm{Cl}}^{s}\right)}^{2}}\right),\; \end{array}$$

where $P_{n}kcc$ is an intensity parameter.

A similar expression can be derived for electrogenic fluxes. The flux across the channel that co-transports one ${\text{Na}}^{+}$ and two ${\text{HCO}}_{3}^{-}$ (nbc) on the stromal membrane can be written as

(10) $$\begin{array}{c} J_{nbc_{s}}=A^{\tilde{s}}P_{nbc_{s}}\left(\ln\left[\frac{C_{\mathrm{Na}}^{c}{\left(C_{{\mathrm{HCO}}_{3}}^{c}\right)}^{2}}{C_{\mathrm{Na}}^{s}{\left(C_{{\mathrm{HCO}}_{3}}^{s}\right)}^{2}}\right]-\phi^{cs}\right),\; \end{array}$$

with a similar expression on the PC membrane,

(11) $$\begin{array}{c} J_{nbc_{p}}=A^{\tilde{p}}P_{nbc_{p}}\left(\ln\left[\frac{C_{\mathrm{Na}}^{c}{\left(C_{{\mathrm{HCO}}_{3}}^{c}\right)}^{2}}{C_{\mathrm{Na}}^{p}{\left(C_{{\mathrm{HCO}}_{3}}^{p}\right)}^{2}}\right]-\phi^{cp}\right).\; \end{array}$$

Antiporters or exchangers, transport one solute species into the cell and another one out of it. For $ae_{s}$ at the stromal side we may write

(12) $$\begin{array}{c} J_{ae_{s}}=A^{\tilde{s}}P_{ae_{s}}\ln\left(\frac{C_{\mathrm{Cl}}^{s}C_{{\mathrm{HCO}}_{3}}^{c}}{C_{\mathrm{Cl}}^{c}C_{{\mathrm{HCO}}_{3}}^{s}}\right),\; \end{array}$$

and on the PC side

(13) $$\begin{array}{c} J_{ae_{p}}=A^{\tilde{p}}P_{ae_{p}}\ln\left(\frac{C_{\mathrm{Cl}}^{p}C_{{\mathrm{HCO}}_{3}}^{c}}{C_{\mathrm{Cl}}^{c}C_{{\mathrm{HCO}}_{3}}^{p}}\right).\; \end{array}$$

Similarly, for nhe, we have the following expression

(14) $$\begin{array}{c} J_{n}he=A^{\tilde{s}}P_{n}he\ln\left(\frac{C_{H}^{s}C_{\mathrm{Na}}^{c}}{C_{H}^{c}C_{\mathrm{Na}}^{s}}\right).\; \end{array}$$

#### ${\text{Na}}^{+}$ -${\text{K}}^{+}$ ATPase

The ${\text{Na}}^{+}$-${\text{K}}^{+}$ ATPase pump transports three ${\text{Na}}^{+}$ out of the cell and two ${\text{K}}^{+}$ in, both against their concentration gradient. Because the energy levels required to fuel the pump significantly exceed the free energy of the system, near-equilibrium thermodynamics cannot be invoked to model the ${\text{Na}}^{+}$-${\text{K}}^{+}$ ATPase, in contrast with cotransporters and antiporters. Therefore, we follow the empirical approach proposed by Strieter et al.,^37^ according to which

(15) $$\begin{array}{c} J_{p}ump=A^{\tilde{p}}P_{p}ump{\left(\frac{C_{\mathrm{Na}}^{c}}{K_{\mathrm{Na}}+C_{\mathrm{Na}}^{c}}\right)}^{3}{\left(\frac{C_{K}^{p}}{K_{K}+C_{K}^{p}}\right)}^{2},\; \end{array}$$

where $P_{p}ump$ (mol/m${}^{2}$/s) is an intensity parameter and $K_{\mathrm{Na}}$ and $K_{K}$ are apparent dissociation constants, which have the following expressions $K_{\mathrm{Na}}=0.2\left(1+C_{K}^{c}/\left(8.33\;\text{mM}\right)\right)$ mM and $K_{K}=0.1\left(1+C_{\mathrm{Na}}^{p}/\left(18.5\;\text{mM}\right)\right)$ mM.^37^

Expressions (9)–(15) are captured in general in the main text in Equation (2).

For every solute i, the overall flux across the cell membrane is the sum of all fluxes through the channels, transporters and exchangers that i is involved with. For example, ${\text{Na}}^{+}$ is transported across the stromal side of the membrane by nkcc and nbc${}_{s}$ (both positive if directed out of the cell) and nhe (positive for ${\text{Na}}^{+}$ if directed into the cell); therefore, the overall ${\text{Na}}^{+}$ flux from cell to stroma can be written as follows

(16) $$\begin{array}{c} J_{1}^{cs}=J_{nkcc}+J_{nbc_{s}}-J_{nhe}.\; \end{array}$$

${\text{Na}}^{+}$ is transported through the PC membrane by pump, which transports three ${\text{Na}}^{+}$ ions out of the cell (therefore, the flux in (15) is multiplied by a factor of 3) and nbc${}_{p}$ (positive is directed out of the cell), hence the flux of ${\text{Na}}^{+}$ from the cell into the PC reads

(17) $$\begin{array}{c} J_{1}^{cp}=3J_{pump}+J_{nbc_{p}}.\; \end{array}$$

The expressions for other ions and membranes can be written analogously, based on the channels reported in Figure 2 and equations reported in this section.

#### Reactions

The terms $R_{i}^{c}$ and $R_{i}^{p}$ in Equations (6a) represent source and sink terms owing to the chemical reactions among species; see Equation (4). They are modeled via the mass action law,^35^ and their expressions read

(18) $$\begin{array}{c} \begin{array}{c} R_{4}^{m}=\left(k_{-1}C_{7}^{m}-k_{1}C_{4}^{m}C_{5}^{m}\right)W^{m},\\ R_{6}^{m}=\left(-k_{h}C_{6}^{m}+k_{d}C_{7}^{m}\right)W^{m},\\ R_{7}^{m}=-R_{6}^{m}-R_{4}^{m}, \end{array}\; \end{array}$$

where m∈{c,p}, $W^{m}$ denotes the volume of compartment m and all other $R_{k}^{m}$ equal to zero. For the PC, $W^{p}=A^{PC}H$ and for the cell, $W^{c}=A^{\tilde{s}}L$.

### Detailed Summary of Equations

We now rewrite the Equations (6a) using the expressions for the fluxes as reported. We also introduce some simplification based on the fact that the reaction (4) is very fast.

For ions ${\text{Na}}^{+}$, ${\text{K}}^{+}$ and ${\text{Cl}}^{-}$ (k=1,2, and 3) the flux balance equations in the cell and the PC are written as:

(19a) $$\begin{array}{ccc} & & 3J_{pump}+J_{nkcc}+J_{nbc_{s}}+J_{nbc_{p}}-J_{nhe}=0\\ & & \;{\text{Na}}^{+}\;\text{flux}\;\text{balance}\;\text{in}\;\text{the}\;\text{cell}, \end{array}$$

(19b) $$\begin{array}{ccc} & & 3J_{pump}+J_{nbc_{p}}+J_{1}^{sp}-QC_{1}^{p}=0\\ & & \;{\text{Na}}^{+}\;\text{flux}\;\text{balance}\;\text{in}\;\text{the}\;\text{PC}, \end{array}$$

(19c) $$\begin{array}{ccc} & & J_{2}^{cp}-2J_{pump}+J_{2}^{cs}+J_{nkcc}=0\\ & & \;{\text{K}}^{+}\;\text{flux}\;\text{balance}\;\text{in}\;\text{the}\;\text{cell}, \end{array}$$

(19d) $$\begin{array}{ccc} & & -2J_{pump}+J_{2}^{cp}+J_{2}^{sp}-QC_{2}^{p}=0\\ & & \;{\text{K}}^{+}\;\text{flux}\;\text{balance}\;\text{in}\;\text{the}\;\text{PC}, \end{array}$$

(19e) $$\begin{array}{ccc} & & J_{3}^{cp}+2J_{nkcc}-J_{ae_{p}}-J_{ae_{s}}=0\\ & & \;{\text{Cl}}^{-}\;\text{flux}\;\text{balance}\;\text{in}\;\text{the}\;\text{cell}, \end{array}$$

(19f) $$\begin{array}{ccc} & & J_{3}^{cp}-J_{ae_{p}}+J_{3}^{sp}-QC_{3}^{p}=0\\ & & \;{\text{Cl}}^{-}\;\text{flux}\;\text{balance}\;\text{in}\;\text{the}\;\text{PC}. \end{array}$$

Note that the superscript sp in ion fluxes stands for the fluxes from stroma into the PC through the tight junctions. We also simplify the rest of the equations by using the fact that the second step of the reaction (4) happens instantaneously.^20^ This means that the equations for k=4,5 can be replaced by a conservation of charge, which is obtained by subtracting Equation (6a) for k=5 from Equation (6a) for k=4 and reads

(19g) $$\begin{array}{ccc} & & J_{ae_{s}}+J_{ae_{p}}+2J_{nbc_{p}}+2J_{nbc_{s}}-J_{nhe}=0\\ & & \;\text{conservation}\;\text{of}\;\text{charge}\;\text{in}\;\text{the}\;\text{cell}, \end{array}$$

(19h) $$\begin{array}{ccc} & & J_{ae_{p}}+2J_{nbc_{p}}+J_{4}^{sp}-J_{5}^{sp}-Q\left(C_{4}^{p}-C_{5}^{p}\right)=0\\ & & \;\text{conservation}\;\text{of}\;\text{charge}\;\text{in}\;\text{the}\;\text{PC}, \end{array}$$

and quasi-equilibrium of the reaction $R_{4}^{m}=0$,

(19i) $$\begin{array}{c} C_{7}^{c}=K_{d}C_{4}^{c}C_{5}^{c}\;\;\;\text{in}\;\text{the}\;\text{cell}, \end{array}$$

(19j) $$\begin{array}{c} C_{7}^{p}=K_{d}C_{4}^{p}C_{5}^{p}\;\text{in}\;\text{the}\;\text{PC}. \end{array}$$

In this expression, $K_{d}=k_{1}/k_{-1}$ is the equilibrium constant, the choice of which is described in the next section. Furthermore, the equation for ${\text{H}}_{2}{\text{CO}}_{3}$ (k=7) is replaced by the conservation of total ${\text{CO}}_{2}$, obtained by summing up Equations (6a) for k=5, k=6, and k=7 and read

(19k) $$\begin{array}{c} \;\;\;\;\;\;\;\;\;\;\begin{array}{c} J_{7}^{cp}+J_{6}^{cp}+J_{7}^{cs}+J_{6}^{cs}+J_{nhe}=0\;\;\text{in}\;\text{the}\;\text{cell}, \end{array} \end{array}$$

(19l) $$\begin{array}{c} \;\;\;\;\;\;\;\;\;\begin{array}{c} J_{7}^{cp}+J_{6}^{cp}+J_{7}^{sp}+J_{6}^{sp}+J_{5}^{sp}\;-\\ \;Q\left(C_{5}^{p}+C_{6}^{p}+C_{7}^{p}\right)=0\;\;\text{in}\;\text{the}\;\text{PC}. \end{array} \end{array}$$

Finally, the equations for ${\text{CO}}_{2}$ (k=6) read

(19m) $$\begin{array}{ccc} & & J_{6}^{cp}+J_{6}^{cs}+W^{c}\left(k_{h}C_{6}^{c}-k_{d}C_{7}^{c}\right)=0\\ & & \;{\text{CO}}_{2}\;\text{flux}\;\text{balance}\;\text{in}\;\text{the}\;\text{cell}, \end{array}$$

(19n) $$\begin{array}{ccc} & & J_{6}^{cp}+J_{6}^{sp}-C_{6}^{p}Q-W^{p}\left(k_{h}C_{6}^{p}-k_{d}C_{7}^{p}\right)=0\\ & & \;{\text{CO}}_{2}\;\text{flux}\;\text{balance}\;\text{in}\;\text{the}\;\text{PC}. \end{array}$$

These equations are solved along with electroneutrality Equations (6c) and water balance Equations (6b), which we rewrite here for the sake of clarity.

(19o) $$\begin{array}{c} \;\;\;\;\;\sum_{i=1}^{7}z_{i}C_{i}^{c}+z_{X}C_{X}=0\;\text{electroneutrality}\;\text{in}\;\text{the}\;\text{cell}, \end{array}$$

(19p) $$\begin{array}{c} \sum_{i=1}^{7}z_{i}C_{i}^{p}=0\;\text{electroneutrality}\;\text{in}\;\text{the}\;\text{PC}, \end{array}$$

(19q) $$\begin{array}{c} Q^{sc}-Q^{cp}=0\;\text{water}\;\text{flux}\;\text{balance}\;\text{in}\;\text{the}\;\text{cell}, \end{array}$$

(19r) $$\begin{array}{c} Q^{cp}+Q^{sp}-Q=0\;\text{water}\;\text{flux}\;\text{balance}\;\text{in}\;\text{the}\;\text{PC}. \end{array}$$

We thus have 18 nonlinear algebraic equations. The unknowns are the concentrations of solutes in the cell and the PC (7+7), the potential in the cell and the PC (1+1), the concentration of fixed non-diffusible charged solutes in the cell (1) and water flux (1).

## Appendix B Appendix 2

### Estimation of Parameter Values

The model relies on a number of parameters, some of which are difficult to estimate, owing to the sparse availability of experimental measurements. In this section, we explain the parameters reported in the Table 1 using the markers from the table sections for easier referencing.

GP We consider that the cell has length of L=10 µm and the PC has length $H=6\cdot 10^{-3}$ m. The area of the PC at the boundary with ciliary processes is estimated to be $A^{PC}=2.6\cdot 10^{-5}$ m${}^{2}$.^4^ Owing to the extensive folding the area of the ciliary processes is considered to be $A^{\tilde{p}}=A^{\tilde{s}}=6\cdot 10^{-4}$ m${}^{2}$.^38^ Note that even though cell membrane at the posterior side is highly folded, this folding is accounted for in values of permeabilities to ions and water instead of the area. The area of the tight junctions is assumed to be 1000 times smaller than that of the whole epithelium owing to the small cleft to cell thickness ratio of the order $O(10^{-3})$.
P-H2O Experimental estimates of hydraulic conductivity of the CE vary from $1.1\cdot 10^{-11}$ m/s/Pa^39^ to 6.26–9.5$\cdot 10^{-11}$ m/s/Pa.^16^ In this work, we set $K^{\tilde{s}}=2\cdot 10^{-11}$ m/s/Pa for the stromal membrane. The NPE basal membrane is highly folded; we account for this folding by increasing its permeability to water and ions by a factor 10, so that $K^{\tilde{p}}=10K^{\tilde{s}}$.
RR The rates of the reactions in (4) for hydration and dehydration of ${\text{CO}}_{2}$ (catalyzed) are taken to be $k_{h}=1.45\cdot 10^{3}$ 1/s and $k_{d}=4.96\cdot 10^{5}$ 1/s (note that a fixed water concentration is incorporated in $k_{h}$).^40^ In the presence of CA inhibition, these rates are reduced by a factor $10^{-6}$. The equilibrium constant of ${\text{H}}_{2}{\text{CO}}_{3}$ dissociation, $K_{d}=k_{1}/k_{-1}=5.3$ 1/mM.^34^
P-C Itel et al.^41^ suggested that permeability of the cell membrane to ${\text{CO}}_{2}$ can range from about $10^{-4}$ m/s to $10^{-2}$ m/s. In our model we impose $P_{{\text{CO}}_{2}}^{\tilde{s}}=1.5\cdot 10^{-3}$ m/s, following the value used in Krahn and Weinstein,^20^ for the proximal tubule brush border. The permeability to ${\text{H}}_{2}{\text{CO}}_{3}$ is also taken from the same article, to be $P_{{\text{H}}_{2}{\text{CO}}_{3}}^{\tilde{s}}=1.28\cdot 10^{-5}$ m/s.^20^ The permeabilities of the posterior membrane to both ${\text{CO}}_{2}$ and ${\text{H}}_{2}{\text{CO}}_{3}$ are assumed to be 10 times larger owing to the membrane folding.
P-ions The value of ion channel permeabilities are hard to estimate, owing to very sparse existing experimental measurements. The model uses as baseline the values reported in Table 1, which were carefully selected to match measured ion concentrations and fluxes and the transepithelial potential difference. We list the parameters that we took from experiments for easier referencing.

[$P_{pump}$]: ${\text{Na}}^{+}$ current density was measured to be approximately 9 µA/cm${}^{2}$,^18^ and a value of 0.22 µEq/h/cm${}^{2}$ for the net ${\text{Na}}^{+}$ flux was found in isolated cat ciliary body.^29^ Thus, ${\text{Na}}^{+}$ flux expressed using the international system of units is in the range of approximately $\left[6.11,9.32\right]\cdot 10^{-7}$ mol/m${}^{2}$/s. These values might be underestimated (see the Discussion), and we have selected the magnitude of the ${\text{Na}}^{+}$-${\text{K}}^{+}$ ATPase to be $6\cdot 10^{-6}$ mol/m${}^{2}$/s.

[$P_{{\text{Cl}}^{-}}^{\tilde{p}}$]: $J_{Cl}^{net}$ is measured to be approximately $\left[3,8.23\right]\cdot 10^{-6}$ Eq/s/m${}^{2}$ from stroma to aqueous.^30^^,^^29^ Using the experimental data reported in Table 2, we estimate the range of permeability to ${\text{Cl}}^{-}$ of the posterior membrane to be within the range $P_{{\text{Cl}}^{-}}^{\tilde{p}}\in \left[2.97,8.16\right]\cdot 10^{-8}$ m/s.

[$P^{tj}$]: Assuming that tight junctions are the main players in determining the value of transepithelial resistance, TER=0.6 kΩ·cm${}^{2}$,^18^ we compute ${\text{Na}}^{+}$ flux through the tight junctions, with the values of concentration from Table 2, to obtain $3.37\cdot 10^{-6}$ mol/m${}^{2}$/s (directed toward stroma). We use this value to estimate the permeability of tight junctions to ions, $P^{tj}=9.22\cdot 10^{-7}$ m/s. A higher value of 6$\cdot 10^{-6}$ m/s was used in Table 1, because it provided the prediction of TEP closer to the experimentally measured value.

Other The temperature considered is T= 310 K and the reflection coefficient for the ions in Equation (5) is taken to be σ=1.^42^^,^^43^ The average charge and valence of fixed non-diffusible species X in the cell is taken to be $z_{X}=-1.5$.
